# Crude Oil and Dispersant Cause Acute Clinicopathological Abnormalities in Hatchling Loggerhead Sea Turtles (*Caretta caretta*)

**DOI:** 10.3389/fvets.2019.00344

**Published:** 2019-10-15

**Authors:** Craig A. Harms, Patricia McClellan-Green, Matthew H. Godfrey, Emily F. Christiansen, Heather J. Broadhurst, Céline A. J. Godard-Codding

**Affiliations:** ^1^Department of Clinical Sciences and Center for Marine Sciences and Technology, College of Veterinary Medicine, North Carolina State University, Morehead City, NC, United States; ^2^Department of Biological Sciences and Center for Marine Sciences and Technology, North Carolina State University, Morehead City, NC, United States; ^3^North Carolina Wildlife Resources Commission, Beaufort, NC, United States; ^4^Nicholas School of the Environment, Duke University Marine Lab, Beaufort, NC, United States; ^5^The Institute of Environmental and Human Health, Texas Tech University and TTU Health Sciences Center, Lubbock, TX, United States

**Keywords:** *Caretta caretta*, corexit, crude oil, dispersant, hematology, loggerhead sea turtle, plasma biochemistry

## Abstract

Following the explosion of the *Deepwater Horizon* MC252 oil rig in 2010, 319 live sea turtles exposed to crude oil and oil-dispersant (Corexit) combinations were admitted to rehabilitation centers for decontamination and treatment. Treatment of oiled sea turtles was guided by expected physiological and pathological effects of crude oil exposure extrapolated from studies in other species and from a single loggerhead sea turtle (*Caretta caretta*) study. While invaluable starting points, inherent limitations to extrapolation, and small sample size of the experimental exposure study, reduce their utility for clinical guidance and for assessing oil spill impacts. Effects of dispersants were not included in the previous experimental exposure study, and cannot be effectively isolated in the analysis of field data from actual spills. A terminal study of pivotal temperature of sex determination using eggs salvaged from doomed loggerhead nests provided an opportunity for an ancillary exposure study to investigate the acute effects of crude oil, dispersant, and a crude oil/dispersant combination in sea turtle hatchlings. Eggs were incubated at 27.2–30.8°C, and hatchlings were randomly assigned to control, oil, dispersant, and combined oil/dispersant exposures for 1 or 4 days. Contaminant exposures were started after a 3 day post-hatching period simulating nest emergence. Turtles were placed in individual glass bowls containing aged seawater and exposed to oil (Gulf Coast—Mixed Crude Oil Sweet, CAS #8002-05-9, 0.833 mL/L) and/or dispersant (Corexit 9500A, 0.083 mL/L), replicating concentrations encountered during oil spills and subsequent response. Statistically significant differences between treatments and non-exposed controls were detected for PCV, AST, uric acid, glucose, calcium, phosphorus, total protein, albumin, globulin, potassium, and sodium. The principal dyscrasias reflected acute osmolar, electrolyte and hydration challenges that were more numerous and greater in combined oil/dispersant exposures at 4 days. Clinicopathological findings were supported by a failure to gain weight (associated with normal hatchling hydration in seawater) in dispersant and combination exposed hatchlings. These findings can help guide clinical response for sea turtles exposed to crude oil and crude oil/dispersant combinations, and indicate potential impacts on wildlife to consider when deploying dispersants in an oil spill response.

## Introduction

For 87 days following the explosion of the *Deepwater Horizon* (DWH) MC252 oil rig in 2010, an estimated 134 million gallons (500,000 m^3^) of crude oil were released into the Gulf of Mexico, with 1.8 million gallons (7,000 m^3^) of surface and subsurface chemical dispersants (Corexit 9500A and Corexit 9527A) applied in an attempt to mitigate impact (1). A calculated estimate of 402,000 surface-pelagic juvenile sea turtles (Kemp's ridleys *Lepidochelys kempii*, green turtles *Chelonia mydas*, loggerheads *Caretta caretta*, and hawksbills *Eretmochelys imbricata*) were exposed to oil, with about 54,800 likely to have been heavily oiled (2). Turtles admitted to rehabilitation centers for decontamination and treatment numbered only 319 (3). Physiological and pathological effects expected from crude oil exposure used to guide treatment of oiled sea turtles came from extrapolation of studies in other species (4) and from one experimental *in vivo* loggerhead sea turtle oil exposure study (5). Effects reasonably expected from crude oil exposure include gastrointestinal pathology, adrenal, and salt gland dysfunction, kidney, and liver pathology, anemia, increased white blood cell (WBC) counts, pneumonia and skin sloughing (4, 5). While invaluable starting points, inherent limitations to extrapolation and small sample size of the loggerhead study limit utility for clinical guidance and for assessing oil spill impacts. For instance, some expected abnormalities such as hemolytic anemia and extensive skin sloughing previously reported in sea turtles exposed to crude oil were not observed in the case of the DWH spill (3, 6). Subsequent to the DWH oil spill, clinicopathological effects on spill-exposed sea turtles have been published from the Canary Islands (7) and from the DWH spill itself (3, 6) that provide further insights on spill impacts to sea turtles. Dispersants, however, were not included in the previous sea turtle oil exposure study, were not a component of spill exposures in the Canary Islands, and cannot be effectively isolated in the analysis of field data from the DWH incident. The combined effects of crude oil and dispersant on sea turtles have not previously been documented.

A terminal study of pivotal temperature of sex determination using loggerhead eggs salvaged from doomed nests provided a rare opportunity for an ancillary exposure study to investigate the acute effects of crude oil, dispersant, and a crude oil/dispersant combination in sea turtle hatchlings. The impact of oil and other environmental pollution in reptiles, and particularly in sea turtles, is little understood due to a scarcity of toxicity data in this taxon (8). Here, we present findings that will prove useful for guiding diagnostics and therapeutics of sea turtles in future oil spills, and inform decisions on whether or not to deploy chemical dispersants.

## Materials and Methods

This study was conducted under endangered species permit 13ST50 from the North Carolina Wildlife Resources Commission, with federal authority delegated from the U.S. Fish and Wildlife Service, and with approval of the North Carolina State University Institutional Animal Care and Use Committee (11-078-O, 11-103-O).

Eggs from loggerhead nests destined to fail from timing or position of nesting and invasive species predation (feral hogs) on a barrier island were salvaged and transported for a terminal pivotal temperature study in 2013. Eggs were incubated under controlled conditions of 27.2–30.8°C following previously described methods for the pivotal temperature study. These temperatures bracket previously reported pivotal temperatures for loggerheads (9). Hatchlings came from 3 clutches, ranging from 106 to 136 eggs, with 56–68% hatch success. Prior to hatching, a subset of eggs was assigned to one of eight treatment groups using a pseudo-random number generator (Windows Excel, Microsoft Corporation, Redmond, WA, USA). After emerging from its eggshell, each hatchling entered its predetermined treatment group 3 days post-hatching, simulating the time expected for emergence from the nest (10). The eight treatment groups (*n* = 15 or 16 per group) were exposed to crude oil (Gulf Coast—Mixed Crude Oil Sweet, CAS #8002-05-9, hereafter referred to simply as crude oil or oil), dispersant product Corexit 9500A (Nalco, Sugar Land, TX; hereafter referred to simply as Corexit), crude oil and Corexit combined, and a negative control (sea water only), for 1 or for 4 days. Sample size was limited to available hatchlings in the primary pivotal temperature study for this pilot study. Each treatment and duration represents a different group of turtles. Sampling occurred at only one time point per group, as indicated (1 or 4 days). Exposures were carried out in flat-bottomed glass bowls (19 cm diameter × 7 cm tall) containing 600 mL aged and filtered seawater at a salinity of 36 ppt and 26°C, one turtle per container, in a room set to a 16:8 h light:dark cycle. Crude oil exposure treatment was 0.833 mL/L and Corexit was 0.083 mL/L, replicating concentrations reasonably encountered during oil spills and subsequent response with a recommended ratio of 1:10 Corexit: oil by the Chemical Response to Oil Spills: Ecological Effects Research Forum (11, 12). Calculated thickness of the crude oil layer resulting from 0.5 ml spread over a surface area of 283 cm^2^, assuming uniform distribution, is 0.018 mm. Turtles were not fed, consistent with their residual yolk dependency for the first few days of life (13), and were monitored 6–10 times daily. Hatching and exposure treatments spanned 28 days. Turtles were weighed, and straight carapace length (SCL) and straight carapace width (SCW) were measured at the beginning and at the end of exposures. At the conclusion of exposures, hatchlings were euthanized by decapitation and pithing (14), gross necropsies were performed, and samples were split among multiple planned studies.

Blood (~0.25 ml) was collected directly from cervical vessels into lithium-heparinized tubes (Terumo™ Capiject™, Fisher Scientific, catalog number 22-256536) at the time of euthanasia and processed within 30 min. Blood smears were stained with a rapid blood stain (HemacolorR, EMD Millipore Corp., Bilerica, MA). Packed cell volume (PCV) was determined using a microhematocrit mini-centrifuge (International Medical Assistance, Inc., Indianapolis, IN). A plasma biochemical panel was performed on a clinical analyzer (VetScan, Abaxis, Union City, CA, with an Avian/Reptile Profile Plus reagent rotor) (15). The biochemical panel included aspartate aminotransferease (AST), creatine kinase (CK), uric acid, glucose, calcium, phosphorus, total protein (TP), albumin, globulin (by calculation), potassium, and sodium. Blood smears were evaluated by a single observer (CAH) blinded to treatment. Estimated WBC count was determined by the formula

WBC estimate (/μL) = average WBC per high power field (10 fields) × (objective power)^2^

where the objective power was 40x (and ocular was 10x), and differential counts were determined from counts of 100 cells based on described leukocyte morphology (16).

Statistical analyses were performed using a commercial statistics software package (JMP® Pro 13.2.0, SAS Inc., Cary, NC). Distribution of data was assessed by the Shapiro-Wilk test. Because many data were not normally distributed, non-parametric statistics were employed throughout for uniformity. Results were compared among treatment groups using the Kruskal-Wallis test. If significant differences were detected, multiple comparisons were performed with Wilcoxon rank sums test between treatment and control corresponding to the day of exposure, and between day 4 and day 1 within each treatment. Changes in weight, SCL and SCW before and after exposures were compared within groups by the Wilcoxon matched pairs signed ranks test. Indices of hemolysis, lipemia and icterus, as determined by the VetScan analyzer, were compared among groups by Fisher's exact test, followed by correspondence analysis if a significant difference was detected. An apparent association between plasma calcium and potassium was assessed by Kendall's τ coefficient. An α < 0.05 was accepted as significant.

## Results

Turtles remained active or resting and responsive to stimuli throughout the exposure period. No skin sloughing, skin lesions, or significant internal gross lesions were observed. Oil was present in the GI tracts of all oil-exposed turtles. Results of morphometrics (Table 1), hematology (Table 2), and plasma biochemistry (Table 3) are shown by treatment group. Statistically significant differences between exposure treatment groups and non-exposed controls, and between 1 and 4 day exposure groups within treatments, were detected for weight, estimated WBC, some differential counts, heterophil/lymphocyte (H/L) ratio, PCV, AST, uric acid, glucose, calcium, phosphorus, total protein, albumin, globulin, potassium, and sodium. Plasma calcium and potassium were significantly positively associated (τ = 0.5052, *p* < 0.0001).

**Table 1:** Morphometrics results by treatment groups, before and after exposures.

**Analyte**	**Control** **1 day**	**Control** **4 days**	**Oil** **1 day**	**Oil** **4 days**	**Corexit** **1 day**	**Corexit** **4 days**	**Both** **1 day**	**Both** **4 days**
Initial Weight (g)	18.5 (17.0 to 20.1)	19 (16.9 to 20.9)	18.7 (17.1 to 21.0)	18.9 (17.3 to 21.3)	18.5 (17.1 to 19.7)	19.0 (16.8 to 20.0)	18.9 (17.1 to 20.3)	18.6 (16.8 to 20.1)
Final Weight (g)	19.2 (18.0 to 20.9)	19.8 (18.6 to 21.7)	19.0 (17.9 to 20.7)	20.0 (17.0 to 21.9)	19.0 (17.3 to 20.7)	**19.0** (16.2 to 20.4)^*^	18.9 (17.5 to 20.6)	**18.2** (16.4 to 20.1)^*^
Δ Weight (g)	0.8 (−0.5 to 1.8)	1.1 (−0.1 to 1.9)	0.3 (−0.7 to 1.3)	1.2 (−1.1 to 2.2)	0.8 (−0.6 to 1.6)	***−0.4*** (−1.0 to 1.4)^*^+	**0.0** (−0.6 to 0.8)^*^	**−0.3** (−1.2 to 0.7)^*^
Initial SCL (mm)	45.8 (43.4 to 47.7)	45.7 (42.7 to 47.4)	46.1 (44.6 to 48.2)	46.9 (42.7 to 48.2)	45.7 (42.7 to 48.2)	46.2 (42.2 to 47.1)	45.8 (42.3 to 47.4)	46.0 (42.5 to 47.5)
Final SCL (mm)	46.2 (44.2 to 48.4)	46.9 (43.3 to 48.0)	45.8 (44.5 to 48.4)	47.3 (43.3 to 49.6)	46.3 (43.4 to 47.5)	46.4 (42.4 to 47.8)	45.9 (42.3 to 47.7)	46.2 (42.4 to 48.0)
Δ SCL (mm)	0.40 (−0.16 to 1.1)	0.46 (−0.12 to 2.2)	0.29 (−0.30 to 0.62)	0.35 (0.05 to 1.60)	0.30 (−0.38 to 1.04)	0.22 (−0.50 to 1.00)	0.33 (−0.60 to 1.16)	0.06 (−0.23 to 1.24)
Initial SCW (mm)	35.3 (34.0 to 37.1)	35.2 (33.2 to 37.2)	35.8 (34.6 to 36.9)	35.8 (31.8 to 37.3)	35.6 (32.5 to 37.0)	35.6 (31.8 to 37.3)	35.5 (32.0 to 37.2)	35.0 (32.7 to 36.9)
Final SCW (mm)	35.1 (34.4 to 36.3)	36.0 (33.7 to 37.5)	35.7 (33.2 to 37.2)	35.9 (32.1 to 37.4)	35.8 (32.4 to 36.6)	35.6 (32.7 to 36.9)	35.5 (31.7 to 37.1)	35.0 (33.0 to 36.9)
Δ SCW (mm)	−0.44 (−1.21 to 1.53)	0.41 (−0.68 to 2.50)	−0.12 (−1.72 to 1.47)	0.29 (−0.84 to 1.98)	−0.02 (−0.52 to 0.72)	−0.02 (−0.97 to 1.20)	0.01 (−1.18 to 1.12)	−0.06 (−1.68 to 1.20)

Bold and

* *indicate significant difference with the control group for the corresponding day*.

*Italics and + indicate the day 4 group differs significantly from the day 1 group for that treatment*.

*N = 14–16 (12–14 for SCW); median (10–90th percentiles)*.

**Table 2:** Hematological results by treatment groups.

**Analyte**	**Control** **1 day**	**Control** **4 days**	**Oil** **1 day**	**Oil** **4 days**	**Corexit** **1 day**	**Corexit** **4 days**	**Both** **1 day**	**Both** **4 days**
PCV^a^ (%)	25 (19–32)	27 (21–29)	27 (20–33)	28 (22–32)	24 (22–31)	*30* *(22–34)+*	**28** (23–33)^*^	**30** (23–33)^*^
WBC Estimate (×10^3^/μL)	6.1 (3.3–14.2)	*3.7* *(2.5–8.1)+*	**11.0** (4.6–14.6)^*^	**10.1** (7.3–22.6)^*^	5.9 (3.7–13.0)	**10.7** (3.2–21.5)^*^	10.7 (4.1–19.7)	**14.2** (5.4–25.5)^*^
Heterophils (%)	69 (55–86)	67 (47–80)	75 (65–88)	70 (63–80)	70 (53–89)	***80*** (71–92)^*^+	**81** (65–90)^*^	**75** (54–92)^*^
Eosinophils (%)	0 (0–1.0)	0 (0–1.0)	0 (0–0.8)	0 (0–1.3)	0 (0–1.4)	0 (0–1.0)	0 (0–0.3)	0 (0–0.50)
Basophils (%)	0 (0–0)	0 (0–0.4)	0 (0–1.4)	0 (0–0)	0 (0–0)	0 (0–0.5)	0 (0–0)	0 (0–0.5)
Lymphocytes (%)	21 (9–34)	21 (14–40)	**15** (3–28)^*^	20 (10–31)	20 (8–40)	***14*** (5–24)^*^+	**13** (6–20)^*^	*20* *(8–35)+*
Monocytes (%)	9 (3–16)	7 (2–20)	8 (2–22)	10 (2–20)	8 (1–15)	4 (0–12)	6 (1–23)	4 (0–17)
Heterophils (×10^3^/μL)	5.0 (2.3–15.3)	*3.5* *(1.6–7.5)+*	**10.2** (4.5–14.9)^*^	**9.5** (6.1–21.1)^*^	5.0 (2.8–13.5)	***10.3*** (2.8–23.0)^*^+	**10.3** (3.8–20.2)^*^	**12.8** (4.7–26.0)^*^
Eosinophils (×10^3^/μL)	0 (0–0.1)	0 (0–0)	0 (0–0)	0 (0–0.2)	0 (0–0.1)	0 (0–0.1)	0 (0–0)	0 (0–0.1)
Basophils (×10^3^/μL)	0 (0–0)	0 (0–0)	0 (0–0.2)	0 (0–0)	0 (0–0)	0 (0–0)	0 (0–0)	0 (0–0.1)
Lymphocytes (×10^3^/μL)	1.7 (0.8–3.3)	1.1 (0.6–2.2)	1.8 (0.3–3.7)	***2.4*** (1.2–5.1)^*^+	1.7 (0.8– 3.3)	1.5 (0.5– 3.3)	1.4 (0.6–2.6)	***3.4*** (1.1–5.2)^*^+
Monocytes (×10^3^/μL)	0.7 (0.2– 1.1)	0.4 (0.1– 1.2)	1.3 (0.2–3.6)	0.9 (0.2–3.4)	0.5 (0.1– 2.0)	0.4 (0.1– 1.4)	0.6 (0.1–4.8)	0.7 (0–3.0)
Heterophil/ Lymphocyte Ratio	3.4 (1.7–10.5)	3.0 (1.2– 5.4)	**4.8** (2.4–28.6)^*^	3.8 (2.0–7.9)	3.5 (1.4–10.2)	***5.8*** (3.1–19.4)^*^+	**6.5** (3.9–13.5)^*^	*4.0* *(1.8–12.6)+*

Bold and

* *indicate significant difference with the control group for the corresponding exposure duration*.

Italics and + indicate the day 4 group differs significantly from the day 1 group for that treatment.

*N = 14–16; median (10–90th percentiles)*.

a *Packed Cell Volume*.

**Table 3:** Plasma biochemical results by treatment groups.

**Analyte**	**Control 1 day**	**Control 4 days**	**Oil** **1 day**	**Oil** **4 days**	**Corexit** **1 day**	**Corexit** **4 days**	**Both** **1 day**	**Both** **4 days**
AST^a^ (U/L)	157 (132–207)	161 (131–206)	186 (129–267)	171 (138–221)	164 (135–225)	***200*** (140–244)^*^+	173 (138–240)	**196** (169–275)^*^
CK^b^ (U/L)	4,234 (2,919–6,153)	3,440 (1,864–6,190)	3,716 (1,222–6,403)	4,496 (2,752–5,852)	4,287 (2,415–7,536)	5,901 (2,408–8,252)	3,432 (1,897–6,996)	5,114 (3,049–8,177)
Uric Acid (mg/dL)	0.7 (0.3–2.1)	0.6 (0.4–1.3)	**1.2** (0.6–4.8)^*^	**1.0** (0.6–2.7)^*^	0.5 (0.3–2.3)	***1.2*** (0.6–3.4)^*^+	**1.3** (0.6–2.6)^*^	***2.5*** (0.7–8.0)^*^+
Glucose (mg/dL)	128 (102–222)	122 (105–149)	129 (105–191)	130 (108–162)	128 (97– 130)	**134** (111–200)^*^	134 (104–212)	***155*** (128–241)^*^+
Ca++ (mg/dL)	8.8 (7.5–10.8)	8.2 (6.4–12.5)	**7.6** (7.3–10.6)^*^	7.8 (6.7–11.0)	9.1 (6.8–11.1)	10.2 (7.4–17.6)	8.0 (7.3–11.8)	9.8 (7.1–17.2)
Phosphorus (mg/dL)	6.8 (5.1–9.5)	*8.0* *(7.2–9.6)+*	7.3 (5.7–10.3)	**8.8** (7.4–12.3)^*^	7.0 (6.2–10.2)	*8.9* *(6.6–11.0)+*	**8.5** (6.6–12.8)^*^	8.4 (6.4–9.9)
Total Protein (g/dL)	2.8 (2.5–3.5)	2.7 (2.4–2.9)	3.0 (2.5–3.9)	2.7 (2.4–3.4)	2.7 (2.5–3.2)	***3.2*** (2.6–3.6)^*^+	**3.1** (2.7–3.6)^*^	**3.3** (2.7–3.8)^*^
Albumin (g/dL)	1.6 (1.4–2.0)	1.5 (1.2–1.8)	1.7 (1.4–2.0)	1.6 (1.4–1.9)	1.5 (1.3–1.8)	1.7 (1.2–2.0)	1.8 (1.4–2.1)	**1.7** (1.1–2.0)^*^
Globulin (g/dL)	1.2 (1.1–1.4)	1.2 (1.0–1.3)	1.2 (1.1–2.0)	1.3 (1.0–1.6)	1.2 (1.1–1.4)	***1.5*** (1.2–1.6)^*^+	**1.4** (1.2–1.7)^*^	**1.5** (1.2–2.3)^*^
K+ (mmol/L)	5.5 (4.7–6.0)	4.8 (3.6–7.0)	5.6 (4.2–7.1)	4.7 (3.9–6.8)	5.3 (4.1–7.0)	***6.6*** (4.1–9.7)^*^+	5.6 (3.8–6.9)	***6.2*** (4.9–9.4)^*^+
Na+ (mmol/L)	162 (148–170)	165 (152–180)	155 (146–169)	156 (149–171)	162 (151–171)	*172* *(157–180)+*	**150** (148–169)^*^	*165* *(149–180)+*

Bold and

* *indicate significant difference with the control group for the corresponding exposure duration*.

*Italics and + indicate the day 4 group differs significantly from the day 1 group for that treatment*.

*N = 14–16; median (10–90th percentiles)*.

a *Aspartate Aminotransferase*.

b *Creatine Kinase*.

Weight increased significantly between initial and final measurements for the control day 1 group (median 4% increase, *p* = 0.0031), the control day 4 group (median 6% increase, *p* = 0.0006), and the Corexit exposure day 1 group (median 4% increase, *p* = 0.0191) but not for the oil exposure day 1 group (median 2% increase, *p* = 0.0753) or day 4 group (median 6% increase, *p* = 0.0561), the Corexit exposure day 4 group (median 2% decrease, *p* = 0.3363), or the combined oil and Corexit day 1 (median 0% change) or day 4 (median 2% decrease, *p* = 0.1140) groups. The SCL increased significantly between initial and final measurements for the control day 1 group (*p* = 0017) the control day 4 group (*p* = 0.0023), the oil exposure day 1 (*p* = 0.012) and day 4 (*p* < 0.0001) groups, the Corexit exposure day 1 group (*p* = 0.0313), and the combined oil and Corexit exposure day 1 group (*p* = 0.0203), but not for the Corexit exposure day 4 group or for the combined oil and Corexit exposure day 4 group. The SCW did not differ between initial and final measurements for any treatment group.

Findings consistent with osmotic, electrolyte, mineral and hydration challenges included (1) significantly higher PCV in combination exposures at days 1 and 4 compared with corresponding controls (Table 2); (2) significantly higher plasma uric acid concentrations in oil exposures at days 1 and 4, Corexit exposures at day 4 (which increased significantly from Corexit at day 1), and combination exposures at day 1 and day 4 (which increased significantly from combination exposures at day 1); (3) significantly lower calcium concentration in oil exposures at day 1; (4) significantly higher phosphorus concentrations in oil exposures at day 4 and combination exposures at day 1; (5) significantly higher TP in Corexit exposures at day 4 (which increased significantly from Corexit exposures at day 1) and combination exposures at days 1 and 4; (6) significantly higher albumin concentration in combination exposures at day 4; (7) significantly higher potassium concentrations in Corexit and combination exposures at day 4 (which increased significantly from their respective exposures at day 1); and (8) significantly lower sodium concentrations in combination exposures at day 1 but sodium concentrations that increased significantly in Corexit and combination exposures on day 4 compared with their concentrations on day 1 (Table 3).

There was no difference in indices of hemolyis (median 0, range 0–2+) or icterus (median 1+, range 0–1+) among groups. There was a significant difference in lipemia index, with the greatest proportion of 1+ lipemia occurring in turtles exposed to both oil and Corexit for 4 days (80 vs. 20% with no lipemia), the next highest proportion of 1+ lipemia being in turtles exposed to oil for 4 days (33%) (*p* < 0.0001). Heinz bodies and morphological changes in WBCs were not observed in blood smears.

## Discussion

### Interpretation of Findings

The principal alterations found in the present study reflect osmotic, electrolyte, mineral, and hydration challenges that collectively were worst in the 4-day combined oil/dispersant exposure treatment. There were additionally indications of a hypothalamic-pituitary-adrenal (HPA) stress response, and potential hepatocellular toxicity. Specifics for each category are addressed in turn, then compared with previous reports. Conclusions from plasma biochemical results are drawn from an in-house clinical analyzer that can vary somewhat from core diagnostic laboratory instruments (15), but performance is consistent and minimal sample volume is required (100 μl whole blood), a distinct advantage when volumes are small and further split for multiple companion studies [e.g., (17, 18)]. Sample size limited power of statistical analysis, particularly with highly variable analytes such as CK and Ca.

Osmotic, electrolyte, mineral, and hydration balance is maintained by the salt gland, kidneys, intestinal epithelium, and skin (19). Fluid loss from any source could result in hemoconcentration, as reflected in increased PCV and TP. The elevations in TP were driven primarily by globulins, which could instead indicate an inflammatory response (although this was not supported by the hematological findings) (16). Albumin determined by the bromocresol green dye binding method (used by the VetScan Analyzer, 15) is less reliable in non-mammalian plasma (20), and globulin is a calculated value from TP and albumin, therefore relative values of these components of TP must be interpreted with caution. Plasma protein electrophoresis is preferred for determining albumin and globulin values in reptile plasma (20), but sample volumes in the present study were insufficient for electrophoresis. Uric acid is the plasma analyte considered to be the best indicator of renal function in reptiles, with even marginal increases in sea turtles serving as a marker of dehydration or renal dysfunction (19). Elevated potassium, sodium, and phosphorus are also consistent with renal dysfunction (16). Potassium may also increase with hemolysis, metabolic acidosis or myopathy (16), but there was virtually no detectable hemolysis and no difference in hemolysis among groups. Acid-base status was not assessed. Phosphorus changes may be dietary in origin, but these turtles were still yolk-dependent and not fed. The lower calcium in day 1 oil exposures is unexplained, but can be seen concurrently with derangements in other electrolytes and minerals (16). It may also represent a statistical anomaly resulting from limited sample size and high variability. Calcium concentrations were also highly variable in other treatment groups, but included some markedly high values (>15 mg/dL) in the Corexit and combined 4 day exposures that could be clinically significant. The lower sodium in combined 1 day exposures also reflects an inconsistency that can be seen with hydration abnormalities in sea turtles (16). This dysregulation could potentially derive from renal, salt gland, or adrenal (via mineralocorticoid) sources. Clinicopathological findings related to hydration challenges were also supported by a failure of hatchlings to gain weight associated with normal hatchling hydration in seawater (21) in Corexit 4 day exposure at and oil and Corexit combination 1 and 4 day exposures. Weight gain of controls was consistent with previously reported hydration-associated weight gain of hatchlings in seawater (21). Increased SCL observed in most groups was associated with normal gradual flattening of the carapace curvature after hatching.

A HPA stress response is suggested by (1) a higher estimated leukocyte count with a higher estimated heterophil count and elevated heterophil/lymphocyte ratio in oil and combined exposures at both time points and Corexit 4 day exposures, (2) higher H/L ratios in oil and combined 1 day exposures and Corexit 4 day exposures (which increased significantly from the 1 day exposure group) (Table 2) and (3) significantly higher glucose in Corexit and combined 4 day exposures compared with controls (which for combined exposure increased significantly from 1 to 4 day exposure groups) (Table 3). An elevated H/L ratio is an indication of a glucocortioid response in multiple taxa (22) that has been evaluated in sea turtles with variable results (23–26). The higher estimated leukocyte and heterophil counts could also have resulted from an inflammatory reaction, however, given that it was not accompanied by a corresponding reduction in lymphocytes. Absence of toxic heterophils and reactive lymphocytes make an inflammatory response less likely. Eosinophils were uncommon in all treatment groups, so reduced eosinophils as a further indication of a glucocorticoid response could not be assessed. Increased plasma glucose is commonly observed as part of an HPA stress response, secondary to increased endogenous glucocorticoids (16). Small sample volumes precluded measurement of plasma corticosterone.

The possibility of hepatocellular toxicity is suggested by higher AST activities for Corexit and combination exposures at 4 days in the absence of differences in CK. Activities for CK, however, were highly variable, and AST elevations were not major.

### Comparison With Prior Studies

Comparing with prior studies of experimental (5) and spill exposures to crude oil (3, 27), several similarities and differences are noted. Differences in exposure conditions and sea turtle life stage likely account for some of the inconsistencies. In the exposure study (5), sample size was even smaller (*n* = 6) than in the current study, limiting statistical power, turtles were older, and larger loggerheads (15–18 mo, 8–12 kg) that would normally be feeding but were fasted, oil thickness at the surface was greater (0.5 vs. 0.018 mm in the present study), and no Corexit was included. In the DWH oil spill exposure (3), animals were primarily pelagic phase Kemp's ridleys and green turtles with only a few loggerheads, larger [0.34–4.0 kg, (28)] but of undetermined ages, degree of exposure was of varying severity and duration for both oil and Corexit, and turtles were evaluated after the additional effects of rescue and extended transport and following through decontamination and rehabilitation. In the Canary Islands, juvenile and subadult pelagic loggerheads (mean weight 9.16 ± 7.74 sd kg) exposed to smaller scale spills from shipping lanes (25) were evaluated as a subset of stranded rescued turtles (7/149) with regards to clinicopathological alterations (27). In this case, exposures were similarly variable, and there was no Corexit exposure. Turtles in these prior studies were well-past yolk-dependence. Clinical chemistry analyzers differed among studies, which can also affect results (15).

In both experimental (5) and spill exposures of sea turtles to crude oil (3, 27), there were indications of anemia. However, in the experimental exposure (5), blood was collected in EDTA, which can artifactually lyse red blood cells of sea turtles, and although erythrocyte counts appeared to decrease, PCV did not, which is inconsistent with a true anemia (3). In spill exposures, anemia may have been associated with comorbidities such as trauma, pneumonia, or gastrointestinal disease (6), some of which could have been independent of oiling, or that developed as later sequelae of oiling. In the case of the DWH oil spill, onset of anemia was variable, and was non-hemolytic, in contrast with previous findings in sea birds that develop hemolytic anemia (3). Additionally, PCV at the time of rescue was highly variable, and included values as high as 47% (6), suggesting at least some turtles experienced hemoconcentration. In the current study, PCV was higher in exposures than in controls, consistent with hemoconcentration, and there was likewise no indication of hemolysis. Anemia may have developed following longer term exposures.

Initially elevated electrolytes and uric acid were commonly observed in sea turtles rescued in the DWH spill (3), consistent with findings in the current study, and with osmotic, electrolyte, and hydration challenges. In the previous experimental crude oil exposure study, authors speculated that there could have been salt gland dysfunction in two of five oil-exposed turtles based on incidental analysis of salt gland secretion, but this was not systematically evaluated and no plasma electrolyte values were presented (5). These plasma electrolyte and uric acid alterations could result from dehydration, or renal, salt gland or adrenal dysfunction. Dehydration might also result from severe epithelial damage, either intestinal or dermal, but in contrast with the previous exposure study and in spill exposure in the Canary Islands in which skin sloughing was prominent (5, 25), no skin sloughing was observed in the current study, and only rarely observed during the DWH spill (6). Presence, absence and degree of skin sloughing in different contexts may result from differences in crude oil composition, weathering, and duration and amount of exposure. For instance, oil depth in the previous crude oil exposure study was 0.5 (5), vs. 0.018 mm in the current study. Lower plasma calcium was observed in oiled turtles in the Canary Islands compared with controls (27), as was hypocalcemia in a number of green turtles following rescue in the DWH spill (6), similar to oil exposure alone at day 1 in the current study. Because sea turtles in the current study were still yolk-dependent, however, malnutrition would not have been responsible for the lower calcium concentrations, as suggested in the field exposure studies. In other treatments in the current study, plasma calcium concentrations were highly variable, but included some markedly high values in some turtles that could be of clinical significance. Both hyper- and hypokalemia were observed in sea turtles rescued in the DWH spill (16), compared with hyperkalemia in Corexit and combined exposures in the current study. A positive association of plasma calcium and potassium, with clinically significant high values of both, is consistent with simultaneously high values reported in a case of sea turtle exertional myopathy (29). In that case it was attributed to cellular degradation, renal dysfunction or both, which could also be factors in oil and dispersant exposure, albeit from different root causes. A companion study of NMR metabolomics using samples from the current exposure study found depletion of the osmolyte taurine in muscle samples that may further reflect osmotic stress of crude oil exposure (18).

Leukocytosis was reported in experimental oil exposure (5) and specifically a heterophilic leukocytosis in spill exposures (3, 27), similar to the higher estimated leukocyte and heterophil counts in the current findings. This was interpreted as most likely a stress leukogram, without ruling out an inflammatory component (3, 5). Over half of turtles rescued in the DWH spill exhibited elevated glucose concentrations, as seen in Corexit and combined exposures at 4 days in the current study, similarly consistent with an HPA stress response. However, hypoglycemia was also observed in several turtles in the DWH response (3, 6), as well as in spill exposures in the Canary Islands (27), attributed to anorexia or exhaustion. Decreases in skeletal muscle lactate and creatinine detected by NMR metabolomic analysis may reflect energy depletion (18), which an HPA response would help mitigate.

Elevated plasma activities of various tissue enzymes (ALP, ALT, AST, CK, LDH) consistent with non-specific tissue damage have been reported in spill exposures (3, 27), but were inconsistently observed and confounded by effects of exertion, rescue, handling, and transport (3). Higher plasma AST activities in Corexit and combined exposures in the current study were minor.

### Clinical Relevance of Findings

Clinical relevance of statistically significant differences in exposure groups may appear minor from a cursory comparison of medians, many of which fall within reference intervals for multiple size classes of loggerheads sampled under varied conditions (16, 30). Some upper 10th percentile values, however, would be of considerable concern for morbidity or potential pending mortality, including uric acid (8.0 mg/dL), potassium (9.7 mmol/L), and phosphorus (12.8 mg/dL). While not statistically significantly different, the same holds true for upper 10th percentile values of plasma calcium (17.7 mg/dL). Plasma uric acid concentrations >2.0 mg/dL are considered problematic in sea turtles (19), and hyperkalemia may lead to fatal cardiac arrhythmias (31). Survival of cold-stunned Kemp's ridleys is lower for turtles presenting with higher plasma uric acid, potassium and phosphorus (32). Plasma uric acid concentration was one of three main determinants of survival in a multispecies model of rehabilitating sea turtles in Taiwan, along with plasma creatinine and presence or absence of a buoyancy disorder (33). Plasma potassium was one of three main determinants in a model of mortality probability for cold-stunned Kemp's ridleys, along with pH and pO_2_ (34). This model was later extended to Kemp's ridleys from the DWH oil spill to calculate that 25% of rescued oiled turtles had physiological derangement putting them at high risk of mortality absent therapeutic intervention (3). No turtles in the current study exhibited a buoyancy disorder, and creatinine, blood pH and pO_2_ were not measured, therefore, neither model could be applied. Nonetheless, even median values of plasma uric acid and potassium in the combined exposure, along with failure to gain weight from normal hydration following immersion, suggest a substantially increased risk of mortality when comparing qualitatively to outcomes in previous studies (32–34).

Results from spill responses indicate that despite dyscrasias associated with oil exposure, there can be a high rate of recovery and release of oiled sea turtles (>88%) if they are rescued, decontaminated and treated (3, 6, 25). However, in the DWH spill, only 574 turtles were rescued and examined, with 319 oiled turtles admitted to rehabilitation centers, out of a calculated estimate of 402,000 surface-pelagic sea turtles that were exposed (2, 3). Approximately 30% of exposed oceanic sea turtles minimally to moderately oiled and 100% of heavily mired turtles are expected to have died (28). More abnormalities were observed with longer (4 days) and combined oil and Corexit exposures in the current study, and these could be expected to exacerbate with more prolonged exposure. Corexit alone after 4 days exposure was associated with more abnormalities than crude oil exposure alone. Combinations of crude oil and Corexit have exhibited enhanced toxicity in a wide range of organisms and cells, including rotifers, corals, fish embryos, juvenile mullet, lymphoproliferative response of dolphins, and others (1, 35–39). Corexit by itself damages epithelia (38), which could include skin, conjunctiva and gastrointestinal epithelia, consistent with observed hydration-related clinicopathological abnormalities. Although skin was not grossly affected in the current study, permeability could have been impacted, as could permeability of the gastrointestinal epithelium. When combined with oil, dispersants increase exposure of organism to crude oil hydrocarbons (38), which could affect renal, adrenal, or salt gland function. Although little to no dioctyl sodium sulfosuccinate (DOSS, used as a marker for Corexit exposure) was detected in turtles that died during the DWH spill, that may have resulted from a relatively high lower limit of quantification, and photo- and bio-degradation in surface waters could have decreased exposure by the time of sampling (40). Corexit was also applied at a lower target dispersant to oil ratio than in the current study [1:20 vs. 1:10 (41)]. Application and exposure were likely heterogeneous, however, and combined exposures early in the event could have persistent effects.

Variability in clinicopathological values of oiled sea turtles within and among studies indicate the need for basic diagnostics on at least a subset of turtles arriving in cohorts during a mass casualty event in order to guide appropriate treatments. This particularly applies to electrolytes, glucose and calcium, in order to tailor fluid therapy. Point of care analyzers requiring only small volumes of blood and yielding rapid results are helpful in this regard (6, 15).

Exposure to crude oil, Corexit, and crude oil-Corexit combined result in osmotic, electrolyte, hydration, HPA and potentially hepatocellular responses and dyscrasias in hatchling loggerheads. These abnormalities were greater and more numerous in 4 day exposures than 1 day exposures, and greater with the combined exposures than single agent exposures. These findings highlight potential hazards to consider when deploying dispersants during an oil spill mitigation response.

## Data Availability Statement

The datasets generated for this study are available on request to the corresponding author.

## Ethics Statement

The animal study was reviewed and approved by North Carolina State University Institutional Animal Care and Use Committee.

## Author Contributions

CH designed the clinicopathological component of the overall exposure study reported here, collected and analyzed samples, analyzed results, and wrote the paper. PM-G conceived the overall exposure study, conducted the exposures, collected and distributed samples to collaborators, and contributed to data analysis and initial writing. MG conducted the pivotal temperature study that provided the opportunity for this ancillary study, acquired the doomed eggs, carried out the egg incubation, collected and processed samples and contributed to writing. EC and HB collected and processed samples and contributed to writing. CG-C conceived the overall exposure study, supported the collection and distribution of samples to collaborators and contributed to writing.

### Conflict of Interest

CH has previously consulted with counsel for British Petroleum on sea turtle health matters. CG-C previously conducted research on sea turtle health following the Deepwater Horizon oil spill event funded from the Natural Resource Damage Assessment Trustees and is currently conducting research on marine mammal health funded by Exxon Neftegas Limited and the Sakhalin Energy Investment. The content herein is solely the responsibility of the authors and does not represent the official views of any past or current funding parties associated with the authors. The remaining authors declare that the research was conducted in the absence of any commercial or financial relationships that could be construed as a potential conflict of interest. The handling Editor declared a past co-authorship with one of the authors CH.
